# Longevity and Plasticity of CFTR Provide an Argument for Noncanonical SNP Organization in Hominid DNA

**DOI:** 10.1371/journal.pone.0109186

**Published:** 2014-10-28

**Authors:** Aubrey E. Hill, Zackery E. Plyler, Hemant Tiwari, Amit Patki, Joel P. Tully, Christopher W. McAtee, Leah A. Moseley, Eric J. Sorscher

**Affiliations:** 1 Department of Computer and Information Sciences, University of Alabama at Birmingham, Birmingham, Alabama, United States of America; 2 Department of Biology, University of Alabama at Birmingham, Birmingham, Alabama, United States of America; 3 Department of Biostatistics, University of Alabama at Birmingham, Birmingham, Alabama, United States of America; 4 Gregory Fleming James Cystic Fibrosis Research Center, University of Alabama at Birmingham, Birmingham, Alabama, United States of America; 5 Department of Medicine, University of Alabama at Birmingham, Birmingham, Alabama, United States of America; Louisiana State University and A & M College, United States of America

## Abstract

Like many other ancient genes, the cystic fibrosis transmembrane conductance regulator (CFTR) has survived for hundreds of millions of years. In this report, we consider whether such prodigious longevity of an individual gene – as opposed to an entire genome or species – should be considered surprising in the face of eons of relentless DNA replication errors, mutagenesis, and other causes of sequence polymorphism. The conventions that modern human SNP patterns result either from purifying selection or random (neutral) drift were not well supported, since extant models account rather poorly for the known plasticity and function (or the established SNP distributions) found in a multitude of genes such as CFTR. Instead, our analysis can be taken as a polemic indicating that SNPs in CFTR and many other mammalian genes may have been generated—and continue to accrue—in a fundamentally more organized manner than would otherwise have been expected. The resulting viewpoint contradicts earlier claims of ‘directional’ or ‘intelligent design-type’ SNP formation, and has important implications regarding the pace of DNA adaptation, the genesis of conserved non-coding DNA, and the extent to which eukaryotic SNP formation should be viewed as adaptive.

## Introduction

The classically hypothesized, random accumulation of single nucleotide polymorphisms (SNPs) through the ages presents a paradox. As a variation on the ratchet mechanism sometimes attributed to Muller [1]–[5] and expanded upon recently by Lynch [6]–[8] and Koonin [9], consider a simplistic estimate that ∼1 in 1000 base pairs from our own genomes have become polymorphic after 150,000 years of human evolution. If, for argument's sake, one were to assume a similar rate of SNP accumulation among older metazoans (omitting, for the moment, the obvious contributions of negative selective pressure and identity by descent) [10], entire genomes would be rendered unrecognizable at every base pairing among vast numbers of ancient genes extant for 150,000,000 years. For still older eukaryotes, the situation would be much worse. This issue has been classically debated, but has not been addressed in the context of specific human genes or the most recent data concerning human DNA. In this report, we apply emerging knowledge from genome scale sequencing projects to view long-term DNA stability in a nontraditional way. An integrated look at a number of quotidian endpoints raises significant questions regarding purifying selection (as well as any sort of evolutionary drift or neutrality [7], [8], [9], [11]) as explanations for the prolonged survival of genes such as CFTR.

In semblance to much of the human genome, CFTR is largely non-coding (total size approximately 190 Kb; cDNA approximately 4500 bp), and like many other human genes has been preserved across diverse species including ancient fish, amphibian, fowl, and mammalian. A great deal is known regarding the genetics and physiology attributable to homozygous or heterozygous CFTR loss in humans. Complete functional absence of one copy of CFTR occurs in 3–4% of American and European Caucasians (over ten million CFTR heterozygotes in North America alone) [12], [13]. Historically, at least one CFTR mutation (F508del) likely conferred a strong selective advantage [14], [15], but no deleterious effect on survival due to a single F508del allele (or any other CFTR mutation) is expected. Phenotypic findings are also absent among mice, pigs, ferrets, and rats deleted for a single CFTR [16]–[19]. In addition, CFTR itself is remarkably flexible and accommodates extensive polymorphism. Homozygous CF (knockout) mice lacking CFTR protein can be restored to health by insertion of a human CFTR different in coding sequence from the murine protein by approximately 30% [16]. CF manifestations can also be reversed in transgenic animals encoding CFTR with a very large (51 amino acid) deletion within the regulatory domain [20].

Mutations in CFTR or any other eukaryotic gene continue to accrue until a threshold of deleterious SNPs is reached, beyond which the profound resilience and plasticity of individual proteins, as well as their crucial epistatic effects (due to multiple loci impacting protein function) will begin to falter. In this report, we argue that over hundreds of millions of years and ongoing SNP accrual, a threshold of this sort should have been expected for CFTR long ago. Note that when individuals or organisms with severe homozygous CFTR defects are culled by purifying selection, this would not overcome a steadily accumulating mutational burden present among surviving contemporaries and their descendants, each being subject to steadily advancing numbers of SNPs over the evolutionary time scale. While overall SNP diversity within a population may fluctuate due to factors such as selection or drift, ongoing accumulation of new DNA variants is very large, and by itself suggests a number of interesting considerations. Population genomics has modeled DNA persistence and stability based on recombination (to reset the mutational ratchet) or a cumulative loss of fitness (attributable to randomly accumulating SNPs and their gene interaction networks) together with natural selection to eliminate detrimental CFTR alleles. Neither of these mechanisms, however, would overcome the continued (and potentially inexorable) accrual of SNPs among surviving members of a population. Our report furnishes recent genomic evidence that SNP accrual over vast numbers of generations could by now have left every CFTR allele so riddled with polymorphism that few, if any, would be viable (regardless of the extent of negative selection), and none would be available to recombine or restore a functional sequence. Moreover, removal of frequent unfit individuals (either an entire species or an occasional cockatrice) by natural selection would not reverse an accumulating CFTR mutational burden within surviving individuals and clades.

Statistical and population-based approaches intended to explain a species averting “mutational meltdown” have not fully addressed emerging knowledge regarding haplosufficiency of vertebrate genes, deep plasticity of the vertebrate genome, the observation that protein coding sequences such as CFTR have been remarkably conserved over hundreds of millions of years (despite an assumption of ongoing SNP accumulation) and new evidence relevant to structure/activity of eukaryotic proteins, including their redundancy and/or expendability. While acknowledging that even a modest decrease in fitness has never been established for the vast majority of random SNPs in any higher eukaryotic gene, an inference has often been that recondite evolutionary pressure somehow holds back SNP accumulation in coding sequences such as CFTR. Because this is a testable hypothesis, we developed our study to address the following questions: 1) In a survey of human populations worldwide using modern and leading-edge genomic tools, including studies conducted to minimize ascertainment bias, what can fundamental patterns of SNP accumulation in CFTR and other critical genes tell us about the production of DNA variants (and particularly the random nature of SNPs at the time of their formation)?, 2) Do these patterns appear to be either spatially or temporally neutral with respect to natural selection?, and 3) Based on a current understanding of CFTR-dependent effects on fitness, what does the analysis indicate regarding the role of purifying selection over the course of human or more ancient hominid evolution? Our findings suggest that modern human SNP compendia are not well reconciled with traditional explanations for long-term DNA persistence (including the role of purifying selection), while at the same time providing no evidence for less conventional (neutral, directional, or intelligent design type [11], [21]–[24]) models of DNA evolution.

## Results

### Analysis of SNP distribution

#### Human exonic and intronic SNP frequencies

We began by tabulating SNP frequency within CFTR and other coding versus non-coding regions of DNA. Our expectation was that SNPs should be less prevalent (e.g., on a per 10,000 nucleotide basis) within the exome; i.e. non-coding DNA can sustain small sequence variations with minimal adaptive consequence [8], [25]–[30]. Assumptions such as these have been challenged to some degree by recent studies indicating up to 80% of the non-coding genome subserves important regulatory function, and that point mutations within ENCODE motifs might often give rise to significant effects on fitness [31]–[33].

Data from dbSNP and HapMap are not optimal for addressing SNP frequency or distribution, since ascertainment bias skews these compendia towards SNPs: 1) discovered previously from selective exonic or other sequencing programs (dbSNP and HapMap), or 2) desirable from the standpoint of hapblock structure; i.e. specifically being sought as ‘informative’ vis-à-vis genome wide or other surveys (HapMap). On the other hand, data from 1000 Genomes provides unbiased and valuable information in this regard. We utilized complete human genomic sequences (approx. 1.8 million SNPs) from an initial 1000 Genomes release (http://pilotbrowser.1000genomes.org/index.html) that were prospective, nonbiased, and manageable in terms of computing.

**Figure 1:**
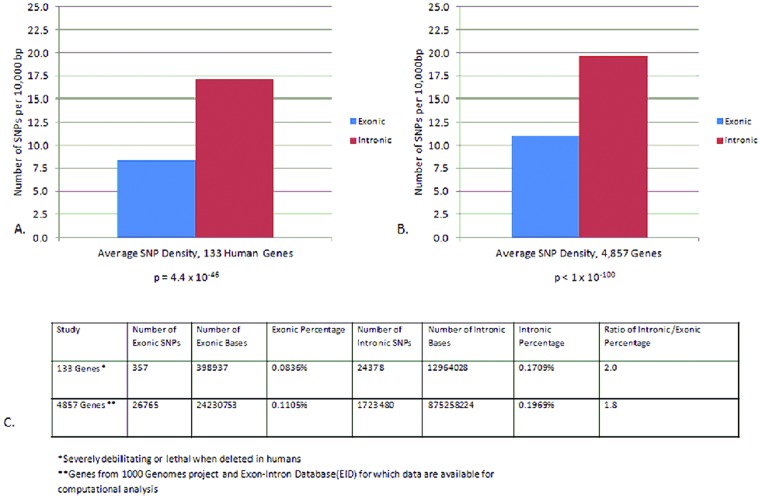
SNP incidence in human intronic and exonic DNA. A: SNPs in 133 human genes known to be lethal or severely debilitating if deleted [90] (Table S1); B: Survey of 4857 human genes for which intron/exon boundaries are readily definable in the Exon-Intron Database (http://www.utoledo.edu/med/depts/bioinfo/database.html) and 1000 Genomes release (http://pilotbrowser.1000genomes.org/index.html); Panel C: Composite data used to generate Panels A and B.

Results in Figure 1 show that overall SNP frequency is diminished in exons versus intronic DNA for CFTR and 132 other genes known to cause serious human illness when disrupted (Figure 1A and Table S1). These genes were chosen because they are expected to be among the most susceptible to intense selective pressure (for many, their homozygous loss being lethal or debilitating). A significant difference in SNP frequency (exon∶intron; 1∶2.0, p = 4.4×10^−46^) was observed when critical human loci shown here were surveyed. A more expansive analysis of 4857 accessible genes indicated a similar ratio of 1∶1.8 (exonic∶intronic).

Is this difference in SNP frequency simply attributable to adaptive purging of deleterious exonic SNPs? If so, it becomes necessary to argue that approximately half of all single nucleotide changes across the human exome (the vast majority of which—including synonymous SNPs—would be of no known functional consequence) were instead highly significant, and that a sizeable number of these (approximately 50%) have been expunged (e.g. due to premature death or decreased fitness). If one accepts the notion that non-coding DNA is also a frequent object of selective pressure (i.e. the ENCODE analysis), even greater numbers of exonic SNPs would need to be removed to account for the findings. In addition, as discussed in detail below, the observation is anything but ‘neutral’ or ‘random’ [11], [21]; the bias in favor of intronic SNPs is robust and appears to occur genome-wide.

#### Enhancement of synonymous versus non-synonymous SNPs in human genes

**Figure 2:**
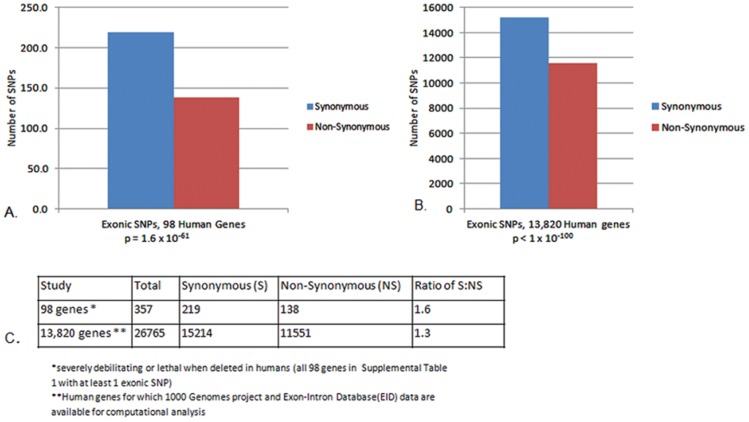
Synonymous and non-synonymous SNP incidence. A: Exonic SNPs in 98 genes known to be lethal or severely debilitating if deleted (a subset of genes in Figure 1A with at least one exonic SNP (Table S2)); B: Survey of 13,820 genes for which data was accessible from the Exon-Intron Database (http://www.utoledo.edu/med/depts/bioinfo/database.html) and 1000 Genomes (http://pilotbrowser.1000genomes.org/index.html); C: Composite data used to generate Panels A and B. All genes from Figure 1A with at least one exonic SNP were examined. Each gene was analyzed in the 1000 Genome Pilot Browser (http://pilotbrowser.1000genomes.org/index.html) including designation as synonymous vs. non-synonymous. The synonymous SNP enhancement agrees with earlier population-based studies in *Drosophila*, human, and other species [35], [36], [37], [84]. To confirm that the ratio of synonymous to non-synonymous SNPs calculated from the set of 98 disease-associated genes was representative of the larger population, a bootstrapping analysis was conducted. Two-thousand samples of 98 genes were randomly selected from the larger gene cohort. Synonymous to non-synonymous ratios were used to determine a mean for each set of ninety-eight chosen in this manner. The overall mean of 2,000 samples was used to calculate both confidence interval and a 2-tailed t-test comparing the means of the 98 disease-associated genes and the mean derived from bootstrap sampling of the larger gene set. At the 95% confidence level, the mean synonymous to non-synonymous ratio of the 13,000 gene data set indicated a ratio between 1.37 and 1.38. A comparison to the 98 gene cohort mean yielded a p-value of 0.12.

Synonymous polymorphisms are often viewed as insignificant from the standpoint of protein function, and are typically disregarded in genome scale studies of human disease (for example, GWAS or somatic SNPs responsible for cancer [27], [28], [34]). Among all genes—and particularly those vital to health—synonymous mutations are much better represented than their non-synonymous counterparts; examples are shown in Figure 2 and Table S2. Synonymous mutations are increased by approximately 1.6 fold among CFTR and ninety-seven other disease-associated genes with at least one exonic SNP. A more extensive test of 13,820 accessible genes (with well-defined exonic-intronic boundaries; Exon-Intron Database, human build 36.1 (http://www.utoledo.edu/med/depts/bioinfo/database.html)) indicated a ratio of 1.3∶1. A similar conclusion has been reached by others in a study based on dbSNP [35] and in exons exhibiting what were presumed to be accelerated rates of evolution [36], [37]. Because a synonymous: non-synonymous ratio of 1∶3—more than reversal of the measured frequency—is anticipated based on random nucleotide replacements within all 64 eukaryotic codons, the data suggests operation of strong selective pressures that have removed deleterious, non-synonymous mutations from human genes. This interpretation, which forms a basis for parallelism as defined by McDonald-Kreitman [22], is considered more fully in a later section.

#### Abundance of transition mutations in CFTR and other human genes

Intronic SNP sequences from CFTR were examined and found to have a relative paucity of transversion (A↔T, G↔C, A↔C, G↔T) in comparison with transition type polymorphisms (T↔C, A↔G, Table 1; *p* = 8.5*×*10^−20^) [10], [38]–[41]. In a 1000 Genomes survey of exonic DNA from CFTR and 97 additional proteins crucial to human health with at least one SNP, a similar pattern was observed. The same was noted when spontaneous (and perhaps more recent) CFTR mutations were analyzed (Table 2) (www.genet.sickkids.on.ca). The transition-favoring aspect has been mechanistically ascribed to a failure of DNA error detection/repair, differences in misincorporation rates, or other factors [10].

**Table 1:** Transition Bias in Human SNPs.

SNP	CFTR Intronic	98 Human genes, Exonic
A/T	16	9
A/G	67 *	150 **
A/C	14	22
G/C	14	29
G/T	16	17
C/T	58 *	130 **

Incidence of six possible SNP configurations (transition and transversion) for CFTR intronic regions, and coding sequence from CFTR and 97 other human genes containing at least one exonic SNP (Figure 2 and Table S2). Underlined = transition mutations. The *p* values (based on an assumption of equal probability for any individual base replacement) indicate a strong bias in favor of transitions over transversions in both the human CFTR intronic DNA and the exonic sequences of 98 human genes. Transition∶transversion ratio for CFTR intronic SNPs = 2.1; for exonic SNPs in 98 genes = 3.6.

*p = 8.5×10^−20^.

**p = 5.7×10^−70^.

**Table 2:** Transition Bias in CFTR Mutations Associated with Human Disease.

Wild Type	Disease associated mutation*	Number of Occurrences	SNP	Total observation
A	C	39		-
C	A	50	A/C	89
A	T	45		-
T	A	57	A/T	102
C	G	49		-
G	C	57	G/C	106
C	T	130		-
T	C	104	C/T	234 #
G	A	179		-
A	G	157	A/G	336 #
G	T	100		-
T	G	69	G/T	169
		Total = 1036		Total = 1036

Incidence of the possible SNP configurations (transition vs. transversion) among>1000 SNPs, many of which have been implicated in clinical CF (http://www.genet.sickkids.on.ca/cftr/app). *p* values indicate a bias towards transition based on an assumption of equal probability for any individual base replacement. Transition:transversion ratio = 1.2.

*http://www.genet.sickkids.on.ca/cftr/app.

**^#^p = 1.3×10^−56^ for transition SNPs.**

#### Founder (ancestral) alleles account for the most common CFTR SNPs among Caucasians

In order to provide context regarding the time frame responsible for appearance of human SNPs shown here, we reviewed haplotype block structure for CFTR and several other genes using HapMap. We utilized data for over 4 million SNPs, drawn from 270 individuals within North American Caucasian (CEU), Han Chinese (CHB), Japanese (JPT), and Yoruba (Nigeria, YRI) ethnic groups.

**Figure 3:**
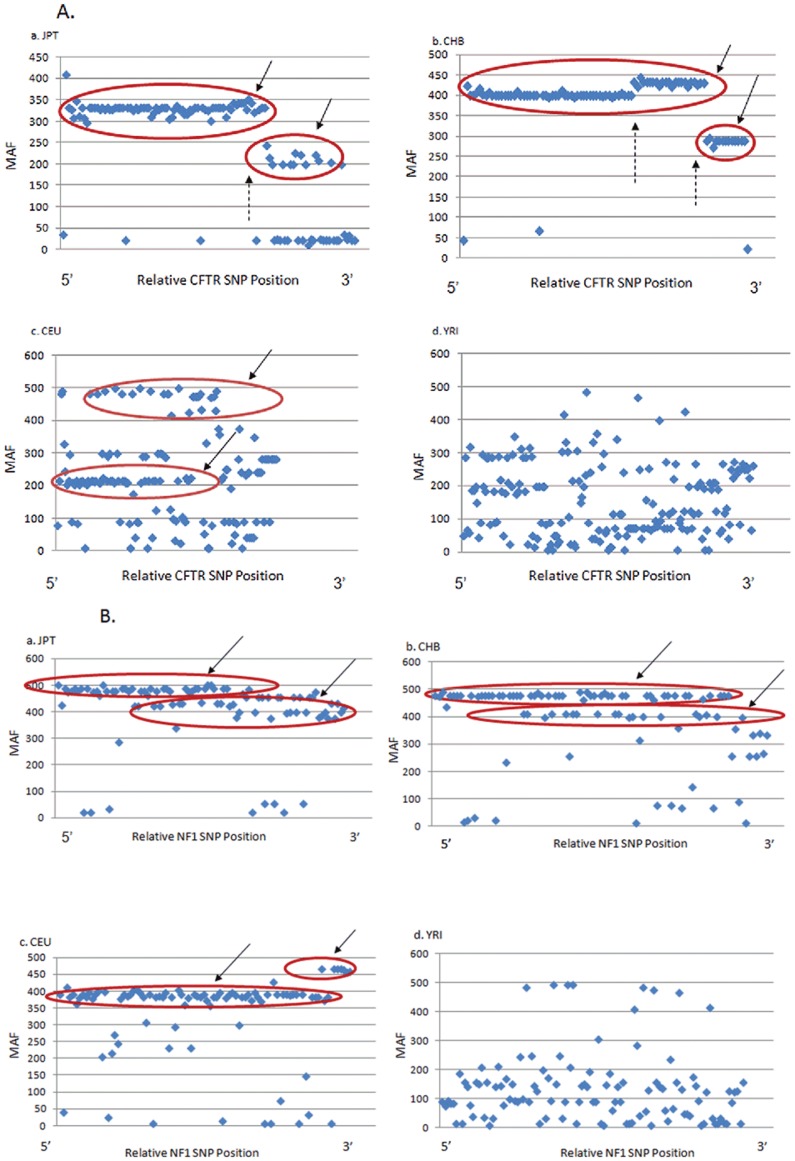
HapMap minor allelic frequencies (MAFs) plotted against gene sequence position. Frequency data for SNPs in CFTR (Panel A) or NF1 (Panel B) were collated for each of the ethnicities shown: JPT (Japanese in Tokyo, 45 individuals); CHB (Han Chinese in Beijing, 45 individuals); CEU (or CEPH, Utah residents with ancestry from northern and eastern Europe, 90 individuals); and YRI (Yoruba in Ibidan, Nigeria, 90 individuals). MAF refers to the relative frequency (1000 = 100% incidence) of the minor allele at each SNP position. Solid arrows/red circles depict areas indicative of a haplotype block (also referred to as MAF block) in the genes as shown; broken arrows describe sites of genomic recombination. In order to generate a MAF block diagram, allele frequency data was downloaded from UCSC genome table browser (http://genome.ucsc.edu/cgi-bin/hg:tables). After downloading, SNPs with MAF equal to zero among all four ethnicities were omitted. The remaining SNPs were then inserted into the scatter plot. Linkage disequilibrium valves for the blocks depicted here (when obtained directly from HapMap) were robust (r^2^ among co∶allelic SNPs shown by red circles typically = 1.0).

Figure 3 and Figure S1 describe incidence of CFTR SNPs from 45 or more subjects per ethnic background. Among JPT and CHB, virtually every SNP (by HapMap intention) is part of a major block, with disruption of the haplotype “clouds” (red circles/solid arrows) occurring due to ancestral crossover events (broken arrows). Because CFTR polymorphisms selected by HapMap were originally identified based on significant frequency in both alleles, there is reasonable agreement between SNPs shown in Figure 3 and those identified by unbiased sequencing in the 1000 Genomes database (i.e. for the specific case of a well-studied gene such as CFTR, approximately 90% of SNPs selected by the HapMap consortium were independently identified by 1000 Genomes). The majority of common CFTR SNPs in HapMap, therefore, are well represented by 1000 Genomes and suitable for the purpose described here.

SNP incidence profiles such as those shown in the CFTR minor allelic frequency (MAF) block diagram (at least for JPT, CHB) obviously cannot be explained by recent mutation in human DNA followed by purifying selection: no MAF block would otherwise be present. Such findings are attributable to ancestral haplotypes—the major source of human polymorphism—with a presumption that large haplotype blocks degenerate over evolutionary time due to recombination [42]. This same interpretation is compatible with the CEU and YRI MAF plots for CFTR, which exhibit degenerating and abolished hapblock structure, respectively (with a caveat regarding the numbers of ancestral or founder haplotypes; see following section). Similar features are shown for NF1, a gene on chromosome 17 that mediates the autosomal dominant disease, neurofibromatosis (Figure 3B).

#### DNA variants on the Y-chromosome further indicate that human SNPs have been contributed in large measure by early ancestral alleles

The Y-chromosome furnishes an independent test of SNP derivation by minimizing contributions of ancestral alleles (anticipated to be reduced by at least 75%, since each ancestral breeding pair contributes four autosomes but only one Y-chromosome; note that if there were only one ancestral male for a specific ethnic group, there would be no SNPs on the Y attributable to founder haplotype). Results from HapMap, dbSNP, or 1000 Genomes are shown in Table 3, and indicate (as reported by others based on a variety of approaches [43]–[48]) a markedly diminished Y-chromosomal SNP incidence. DNA sequencing obstacles, sampling bias, background selection etc., contribute to this finding and the overall, quantitative difference is not known [44], [46], [48]. However, it is clear that Y-chromosomal SNPs are far fewer in number, including those within more readily sequenced regions (Table 3). The data, therefore, support early ancestral haplotypes—rather than ongoing DNA mutation—as a major contributor to SNP distributions among modern humans (i.e. SNPs of identity descent) [10], [42].

**Table 3:** Frequency of SNPs on the Y and other representative human chromosomes.

		Total SNPs			SNPs per 10,000 bp		
Chromosome	Size (bp)	db SNP	Hapmap (CEU)	1000 Genomes*	db SNP	Hapmap (CEU)	1000 Genomes*
Chr:22	49691432	399169	55941	251649	80.33	11.258	50.642
Chr:21	46944323	369905	50983	219897	78.797	10.86	46.842
Chr:20	62435964	623847	121069	396676	99.918	19.391	63.533
Chr:X	154913754	847225	122601	556264	54.69	7.914	35.908
Chr:Y	57772594	50993	722	326	8.827	0.125	0.056
Genes on the Chr Y	2173359	171	85	244	0.787	0.391	1.123

Number of SNPs is given for each of the chromosomes shown, according to data in dbSNP, HapMap, or 1000 Genomes.

*1000 Genomes Pilot Release 7.

Note that if point mutations in CFTR and other human genes were accounted for purely by *de novo* DNA mutation among *Homo sapiens*, rates of Y-chromosomal SNPs should be approx. 50% of autosomal SNP frequency (one Y-chromosome for every two autosomes). Anything less than 50% can be conditionally attributed to founder (ancestral) derived autosomal alleles. Because the measured SNP incidence of the Y (Table 3) is consistently less than 2% of the autosomal SNP frequency, the findings suggest that over 95% of autosomal SNPs could have been contributed by founder haplotypes. Small differences in chromosome-specific mutation rates would not significantly alter this estimate, although background selection may significantly diminish Y chromosome diversity, and complicates analyses of this kind [48].

#### Non-synonymous SNP rates in CFTR and other human genes are lower than expected

Standard SNP frequencies reviewed in Figures 1 and 2 (and associated Tables) agree with findings from many laboratories investigating DNA variants among human and other species, and provide an argument against ‘neutral’ or ‘near neutral’ models of genomic evolution. Otherwise, individual alleles, large segments of the genome, as well as both coding and non-coding DNA would be required to drift in an overwhelmingly biased and uniform direction, and drift (by definition) occurs randomly [11], [21], [23], [27]. Note that neutral models do not exclude specific genomic elements exhibiting only limited variation (e.g. hyperconserved segments), although such intervals are not believed to represent a predominant component of human DNA. By the same token, near-neutral models allow for significant numbers of deleterious variants to become fixed in small populations (i.e., tending to favor non-synonymous SNPs). In this context, therefore, it becomes informative to scrutinize the quantitative significance of a synonymous to non-synonymous SNP ratio of roughly 1.5∶1 (Figure 2).

Consider, for example, a pair of extant Caucasian individuals with a common ancestor 50,000 years ago (an estimated time of hominid migration out of Africa) [49], who now differ at approximately 1 in 1,000 nucleotide positions throughout their respective genomes [50]. If exonic DNA conservatively represents ∼3% of three billion human nucleotide pairs, this amounts to approximately 90 million exonic positions with ∼0.1% rate of single nucleotide polymorphism, or on the order of 90,000 exonic SNPs. The data in Figure 2 indicates that upwards of 54,000 of these should be synonymous, with roughly 36,000 non-synonymous, genome-wide (i.e. synonymous: non-synonymous ratio of ∼1.5).

As introduced above, in the absence of natural selection, the expected ratio of non-synonymous to synonymous polymorphisms on a full genome basis is usually taken to reflect stochastic SNP formation, since factors such as drift, shift, etc. would be minimized by random assortment. A neutral or random accrual of exonic SNPs generates a quantitative ratio of approximately 3∶1 (non-synonymous to synonymous; accounting for all possible nucleotide changes in all possible human codons). The “expected” incidence across 90,000 exonic SNPs (omitting natural selection for the moment) is therefore at least a reversal of the “observed”—i.e. ∼68,000 non-synonymous and ∼22,000 synonymous SNPs. (Codon usage as a means to preserve exonic DNA is considered later in this report.)

Although fitness is an exceedingly difficult variable to quantify [51], it is problematic to imagine that negative selection could lead to such a reversal of the non-synonymous to synonymous SNP ratio over an extended period of human evolution. First, the mutation rate in humans (1–3×10^−8^ SNPs/nucleotide/generation [52]–[55]), and the estimated ∼54,000 synonymous SNPs across ∼90 million exonic positions would be predicted to require>400,000 (not 50,000) years—i.e. most of the known synonymous SNPs must have significantly predated the ethnic founders of modern *Homo sapiens*. This agrees with conclusions from the MAF block analysis shown in Figure 3—i.e. that most human SNPs are not the result of recent DNA mutation, but attributable to ancient ancestral alleles.

Second, and more importantly, the synonymous versus non-synonymous SNP ratios cannot be easily reconciled with what is known about CFTR and many other human gene products. In every eukaryotic genome, SNPs continue to accumulate with each ensuing generation, and at some point would be anticipated to alter fitness. As described above, the “expected” versus “observed” SNP quota requires that under a natural selective pressure, human genomes have evolved from an expected (and stochastic) ratio of 1∶3 (synonymous: non-synonymous) to the observed ratio of ∼1.5∶1. This would indicate that more than three of every four (approx. 78%) non-synonymous SNPs in CFTR and other genes have been deleted (or at least markedly de-enriched) from the gene pool either by classic (purifying) selection, or due to more complex, multigenic effects. The model presents a “best case” scenario, since rare mutations with a positive effect on fitness would require still higher rates of purifying selection to arrive at the same net loss of non-synonymous polymorphism. Note this analysis is quantitative and does not involve statistical or other underlying demographic assumption. (The calculation is based solely on empiric data including the directly measured (and widely accepted) genomic enrichment for synonymous vs. non-synonymous human SNPs, the expected ratio of synonymous: non-synonymous SNPs if formation was random—a value obtained from the genetic code, the best available (and readily quantified) degree of human DNA polymorphism, and size of the human genome.) The available and published findings point to a decrease from ∼68,000 (predicted) to ∼36,000 (observed) non-synonymous SNPs, or roughly 32,000 pre-reproductive age deaths or unfit genomes deleted or severely repressed during a finite period of human (or, in fact, pre-human) evolution.

#### Purifying selection and modern SNP ratios

The assertion that an estimated 32,000 single nucleotide changes (distributed over a genome with 20,000–30,000 genes) have been expunged from the gene pool of two individual humans with a common (pre-human) ancestor highlights the rarity of these mutational events. The notion that an isolated, randomly placed non-synonymous SNP in a diploid gene such as CFTR should not only abrogate function, but also lead to death or blunt fertility of the entire organism seems incompatible with the puissant effects on fitness that would be required to explain the modern SNP distributions reviewed here. Put plainly, it seems antithetical to suppose that a single, random base substitution in any gene should disrupt activity or severely undermine fitness of an entire human, let alone that this has occurred tens of thousands of separate times over the course of an individual's evolutionary descent from an ancestral founder.

As shown in Figure 2, the synonymous to non-synonymous ratio is similar whether 98 (disease-associated) or 13,820 distinct human genes are analyzed (a value of 1.3–1.6). Because the proportion of synonymous to non-synonymous SNPs applies across numerous chromosomes, deleterious effects concentrated in a small number of genes cannot account for the finding. Moreover, if this level of non-synonymous SNP reduction represents a ‘mutational burden’ near to a significant (additive or fractional) effect of mutations on fitness, one could argue that many extant human genes (and individuals) should exist near some critical threshold, limping along and barely able to accommodate further polymorphism. As noted above, this is not the case for modern human CFTR, which appears strongly accommodating to polymorphism. Moreover, the notion that one random point mutation in a single allele of any human gene would usually (in almost 80% of cases) cause death or severely abrogate fitness is at odds with much of what has been learned about human gene and protein plasticity over the past 50 years.

Imagine that technology were available to place one exonic SNP in a single diploid gene randomly in the human genome. The likelihood that this individual SNP would be sufficient to destroy (or render less fertile) an entire individual is remote. Moreover, *Homo sapiens* is a comparatively young species. If this level of polymorphism represents a general threshold (i.e. a “tipping point”) beyond which fitness is lost as genomes decompensate, it is difficult to imagine how a panoply of much more ancient genes (among far more ancient species) could have survived during an evolutionary period thousands of times more prolonged. In addition, note that in recombinant murine models, an extensive database already exists with regard to the same question. As with human, the likelihood that a single (random) non-synonymous SNP per murine gene should be a common cause of infertility or death is diminishingly small based on a vast number of transgenic animals and experimental findings. Yet the data reviewed in Figures 1 and 2 require a remarkable selective pressure of roughly this magnitude in order to account for observed SNP frequencies among the same genes in human DNA (i.e. one of every two random, exonic SNPs (including synonymous SNPs, Figure 1), or just one random, non-synonymous SNP per human gene (Figure 2) appears to be so deleterious that it typically causes death or abrogates normal reproduction).

#### Inurement of DNA polymorphism in the eukaryotic genome

Our earliest hominid ancestors inherited CFTR as part of a genetic legacy hundreds of millions of years old. Irrespective of whether or not modern CFTR SNP patterns are attributable to recent purifying selection, the integrity and flexibility of the human gene is well-established. How did CFTR persist without becoming riddled with polymorphism despite its ancient origins and subsequent epochs of mutation accrual? With regard to a central question posed by this report, consider hominid evolution during the past 200,000 years and a DNA mutation rate (described above) with approx. 90 new SNPs per individual per generation (for review, [46], [51], [56]). During 10,000 generations (assuming 20 years each), an estimated 9×10^5^ mutations would be expected to distinguish a present-day individual from an early hominid ancestor. Now apply this same process for a much longer period (among significantly older metazoans) and, for the moment, omit the role of purifying selective pressure. After 250×10^6^ years (the evolutionary age of many sharks), mutations would be expected in at least every codon of any sizable vertebrate genome; e.g., every codon in a 3×10^9^ base pair genome would be altered in a random fashion. If a more realistic generation time (e.g. one year) is imposed, every codon in every core metabolic gene would be altered 20 times, and in a genome of 30,000,000 bp (perhaps more representative of certain diploid ancestors), each codon in every core metabolic gene would be randomly replaced nearly 2,000 times. Modestly lower rates of human mutation [e.g. 3]-[fold less, compare 53–55, 57] do not substantially alter this analysis. Moreover, a computer simulation conducted by our laboratory demonstrated <6% concordance of modern CFTR versus an ancient ancestor under these conditions, and that the last CFTR with>30% homology to the original CF gene product would have disappeared hundreds of millions of years ago. In other words, regardless of the type and magnitude of selective pressure that might have been applied, no CFTR would be expected today that even remotely resembles a functional protein. Moreover, even if one were to skew the analysis (and impose additional assumptions regarding population size, drift, evolutionary bottlenecks, etc.) so that CFTR somehow survived and retained its plasticity, the likelihood that an individual human with a working copy of CFTR would have also preserved 20,000–30,000 other human genes in exactly the same fashion (each gene having experienced its own stochastic mutational burden over hundreds of millions of years) does not seem compatible with genomic persistence. Regardless of population size, variation of the fitness landscape, putative valleys, drift etc. invoked so that observations better approximate the evolutionary expectation, the analysis strongly indicates that “meltdown” of human CFTR is long overdue.

In summary, based on new and emerging knowledge regarding DNA polymorphism, the classical argument that natural selection or DNA recombination somehow reset the “ratchet” mechanism described above [58]–[60] does not account for significant discrepancy. The mutational burden continues to accumulate towards “meltdown” in every generation and every member of a given population. Even when natural selection removes certain disadvantageous haplotypes or enriches others, all remaining alleles continue to experience an ever-increasing SNP burden through the ages. Recombination, drift, and natural selection cannot stave off the mutational juggernaut, since every allele available for recombining continues to experience its own accumulating mutational burden, and every diploid gene that evades selection will continue to accumulate SNPs. While overall SNP diversity of a human population will fluctuate due to factors such as these, the perpetual accumulation of new DNA variants over an evolutionary time frame is very large. Moreover, even if a specific gene somehow managed to persist, its sequence could be ransacked by extensive polymorphism, and close to the threshold for dissipation. In other words, detrimental fitness effects necessary to overcome evolutionary destruction of CFTR—a eukaryotic gene that like others exhibits remarkable plasticity—account poorly for the extreme longevity of CFTR or the surrounding genome.

#### Concordance between SNPs in human exons and coding sequences from other species

**Figure 4:**
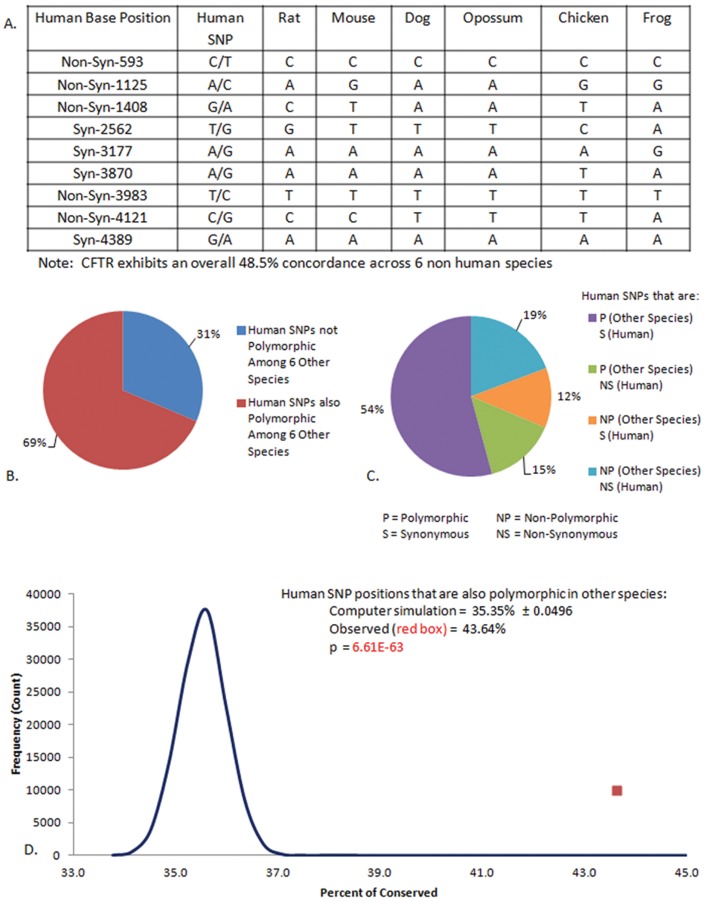
Positions exhibiting polymorphism in human CFTR are also polymorphic among other species. A: Six of nine CFTR coding SNPs identified by unbiased analysis of individuals in 1000 Genomes were also were polymorphic among diverse species, despite approximately 50% overall nucleotide identity among the non-human CFTRs being analyzed. B, C: CFTR and 21 other genes (Table S3) were investigated in the same fashion shown in Panel A. The majority of SNPs in exonic regions found to be polymorphic were synonymous (*p* = 2.7×10^−9^, versus the stochastic ratio otherwise expected for non-synonymous to synonymous polymorphism). In order to increase stringency, only those genes in Figure 2A with ≥50% concordance across the six non-human species were included in the analysis. D. CFTR homologs in four evolutionarily distant species (horse, frog, zebrafish, and shark) were aligned with the human coding strand, both independently and collectively. In the collective alignment, ∼43% of the coding sequence was invariant. A computer simulation was conducted and the total number of differences from human placed randomly within the human CFTR reading frame of 4443 bp. The goal was to determine in a conservative fashion whether concordance observed in a multiple species alignment could be accounted for by chance. The simulation was performed 120,000 times and the numbers of differences from human tabulated. The mean concordance (35.4%) and standard deviation (∼0.05) for this set of simulation data was calculated and differed significantly from the higher level of identity observed in nature for the multiple species alignment (p = 6.6×10^−63^).

We also compared exonic regions in human genes found permissive for SNPs (i.e. “polymorphic” by McDonald-Kreitman criteria; [22], [61]) and the corresponding regions in six other chordates. An example depicting the first nine exonic SNPs reported in CFTR by 1000 Genomes is shown in Figure 4A. The complete CFTR open reading frame is approximately 50% identical among these six non-human CFTRs, including evolutionarily distant species such as chicken and frog. A corresponding but more extensive analysis is shown for CFTR and twenty-one other human genes with ≥50% overall concordance and at least one exonic SNP identified by 1000 Genomes (Table S3). The findings summarized in Figure 4B establish that the same DNA positions exhibiting single nucleotide polymorphism among humans also tend to be concordant with polymorphic sites from evolutionarily distant species. Similar results have been shown previously by others [22], [62], [63]. Notably, DNA positions polymorphic in humans (and the corresponding polymorphic positions among non-human species) are predominantly *synonymous* (Figure 4C; *p* = 2.7×10^−9^). When complete coding sequences from 629 individuals and a recent 1000 Genomes release were analyzed, approx. 69% of positions found to be polymorphic among both humans and multiple other species were synonymous (*p* = 3.2×10^−43^ versus the expected number of synonymous SNPs if accumulation were randomly distributed). Since variability is enriched in a strongly synonymous fashion, these mutations are unlikely to represent DNA positions where SNPs have been established by purifying selection. Moreover, since a robust synonymous bias occurs genome wide, neutrality or drift do not furnish a satisfactory explanation. In a related study, we observed that CFTR exonic concordance among four vertebrate species evolutionarily distant from human (horse, frog, zebrafish, and shark) is approximately 43% and that the total number of single nucleotide differences between these four species and human CFTR is 4620. The preponderance of shared SNPs among the four species was again found to be synonymous. In addition, when a SNP was applied at random in 4620 distinct instances to the 4443 base pair open reading frame of human CFTR using computer simulation, concordance between human and non-human coding sequences was much lower than observed in nature (average ∼35%; *p* = 6.6×10^−63^) (Figure 4D). Observations such as these point to an important question: Why do so many synonymous DNA changes—mutations of questionable adaptive relevance—exhibit significant conservation across numerous species?

## Discussion

Recent studies of CFTR sequence evolution [64], protein residue co-evolution and structure [65], selection for intronic regulatory sequences [33], inferences regarding CFTR channel gating [66], and characterization of the cystic fibrosis disease mutational spectrum [67] are predicated on a mechanism that treats CFTR exonic, intronic, synonymous, and non-synonymous SNP production as a random process. More classical aspects of genomics including DNA ‘clocks’ [28], polymorphic SNP formation and non-neutral evolution [22], [30], [36], rapidly evolving and ultra-conserved DNA [26], genesis of phenotypic complexity [68]–[70], and computational reconstruction of ancestral DNA [71] are likewise grounded to varying extent on SNP formation as essentially unbiased. The present study, however, suggests that inadequate attention has been paid to the non-random features of SNP formation. Based on recent knowledge regarding CFTR (and other protein) plasticity, function, and SNP distribution, our analysis indicates that approximately half of all exonic SNPs and nearly 80% of non-synonymous SNPs that should have been expected on a stochastic basis in human DNA instead were never formed in the first place.

Note that the above statement is by no means meant to imply that evolutionary selection does not purge deleterious mutation. However, insofar as human DNA is concerned, even if one adopts a very conservative estimate that a single SNP anywhere in the ∼90 million nucleotides of human coding DNA has a 1 in 10 chance of causing death or undermining fitness of the entire human organism, we are left with an estimate that among SNPs expected on a random basis, 45% of all exonic SNPs and>70% of non-synonymous SNPs instead were never produced. Below we provide a summary that underscores the topics dealt with by this report.

- Figure 1 and Table S1 establish that introns exhibit a strong increase in SNP frequency whether investigated among a selected gene set or across the entire human genome (a ratio of approx. 2∶1 intronic versus exonic SNPs; *p* = 4.4×10^−46^). The assumption that this results from selective removal of exonic SNPs seems unsatisfactory, since it would require at least one of every two coding SNPs (including a preponderance of synonymous SNPs) to be markedly detrimental, leading to early death or otherwise undermining fertility, irrespective of epistasis (see also below). The likelihood that a solitary, randomly placed exonic SNP in CFTR (or other gene) should be lethal or vitiate fertility is contrasted by a substantial body of modern evidence regarding protein function and plasticity. We believe an alternative explanation has not been adequately considered; namely, that the modern SNP distributions shown here are attributable in large measure to a strong bias in their original formation. (In this context, human SNPs are not ‘neutral’ [13], [16]–[21]; there is a strong and highly significant bias towards non-coding SNPs among individual genes, groups of genes, and genome wide (Figure 1), yet the observation is not directional as described by Cairns [24], [72] or the result of intelligent design [73]).
- Figure 2 describes a strong increase in synonymous SNPs compared to their non-synonymous counterparts (approx. 1.5∶1) when exons are investigated from CFTR, multiple human genes and across the entire genome. Again, purifying selection does not provide a complete or satisfactory explanation, since a natural selective mechanism would require that during our evolutionary past, DNA has been so inexplicably brittle that just one new non-synonymous SNP per diploid gene routinely led to death or interrupted fertility of an entire human ancestor. The requirement that a single, randomly placed SNP would typically have such an effect on CFTR (or any protein) needs to be carefully interpreted. The number of human exonic SNP positions per gene is comparatively small, and it is not satisfactory to imbue these infrequent polymorphisms with such an overwhelming effect on fitness. Again, an alternative explanation seems to imply that synonymous SNPs were produced (at the time of their formation) in a substantially biased fashion, and at much higher frequencies than their non-synonymous counterparts. In this context, when we analyzed complete genomic sequences from 16 different murine strains (from http://www.sanger.ac.uk/cgibin/modelorgs/mousegenomes/snps.pl), heterozygous positions attributable to very recent SNP formation among congenic murine lines removed from many forms of selection (i.e. variants produced in a “minimally selective” laboratory environment with negligible predatory, pathogenic, reproductive, or environmental pressure), we measured a ratio of 1.6∶1 synonymous to non-synonymous substitutions (*p* = 8.3×10^−49^) ([74] and unpublished results). This observation, as with the human data, is best explained by a strong bias favoring synonymous SNPs at the time of formation.
- The data in Figure 4 indicate that positions of human exonic SNPs strongly resemble the corresponding sites of polymorphism among numerous evolutionarily distant species. A classical interpretation that this represents selective removal of the same detrimental point mutations across human and multiple other genomes does not account for the findings, in part because the conserved SNPs are predominantly synonymous. Instead, the results appear to suggest that SNPs across many species have been produced in a fashion that is more biased (or constrained) than classically appreciated. The observation again applies to individual eukaryotic loci such as CFTR, and a survey representing larger cohorts of genes. Examples of **specific mechanisms** that could account for pathways of this type are described later in this report.
- Note that few (if any) studies have considered the possibility that modern human SNP patterns might depend more on the ways mutations were originally produced than the extent to which they have subsequently been selected or randomly fixed. SNP formation bias is typically neglected by current models of both exonic and protein evolution. Yet our data suggest that hominid SNP *formation* has been much more frequent (on a per nucleotide basis) in non-coding (compared to coding) DNA, and that exonic SNPs are far more likely to be created as synonymous (versus non-synonymous) variants.
- Finally, data in other Figures and Tables of the report (and throughout the text) offer a human genomic context for the well-described mutational “ratchet” scenario contemplated years ago by Muller and colleagues. One can argue that over eons of eukaryotic evolution, a mutational burden should (by now) have decimated CFTR and other diploid genes. Yet despite hundreds of millions of years of ongoing mutation accrual in core metabolic proteins, the human genome does not appear to be anywhere near “meltdown.” The notion that spectacular longevity of higher eukaryotic DNA is somehow accounted for by selective elimination of detrimental SNPs does not adequately explain the findings. Although it is clear that individuals (and entire species) are constantly being expunged from the global gene pool, this would have no effect (and would not reverse) ongoing SNP accumulation in the genes or genomes of all surviving individuals (and species) whose burden of DNA mutation is not resolved simply by “weeding out” of others. SNP accrual over countless generations might be expected to leave every allele riddled with polymorphism (regardless of negative selection), and eliminate sequences that could otherwise recombine to restore a functional protein. This dilemma has not been fully considered in light of modern functional genomics; computer simulation indicates “mutational meltdown” should have occurred millions of years ago.

### An alternative hypothesis

Perhaps SNP generation over the evolutionary timescale has been fundamentally less random than we typically assume. Under this arrangement, the major (high MAF) human SNPs in HapMap and 1000 Genomes would have appeared in a fashion configured to prevent overwhelming genomic attrition (for example, with SNP formation directed towards noncoding DNA and synonymous polymorphism). Such a model would help reconcile observations that human SNP populations (both synonymous and non-synonymous) are non-random (and non-neutral) in distribution (Figures 2, 3, 4, Figure S1), yet are not well explained by purifying selection (Figures 1–3; and arguments above regarding the plasticity of human genes, fitness effects necessary to account for the prevalence of non-synonymous variants, measured frequency of synonymous SNPs in human DNA, conserved silent DNA variants among multiple species, magnitude of damage to an entire organism that would be required to purge human SNPs from coding DNA, etc.) [11], [23], [76]–[78]. Moreover, we found no evidence for SNP ‘directionality’ as an adaptive mechanism in specific genes, or ‘intelligent design’ suggested by others [24], [72], [73], [75]. The same SNP patterns were observed across numerous genes and protein functional categories, and the DNA changes were strongly synonymous. In addition, ‘directional’ mutations (suggested to enhance fitness) would not reverse DNA attrition, which is solely a consequence of mutation rate and time.

### Implications: A mechanistic perspective

While “non-randomness” or “formation bias” could be taken prosaically to imply large numbers of irrelevant SNP “hot spots” or other physical factors that contribute to SNP accumulation, we suggest that survival of DNA and establishment of genomic polymorphism are so crucial they might not be left to chance alone [75], [79]–[81]. For example, note that transition mutations within human ancestral alleles are very strongly favored (Tables 1 and 2), and this bias results in an exon conserving effect throughout the genome. We take the transition bias, by itself, as compelling evidence for a robust **mechanism** that opposes genetic dissipation and dictates patterns of SNP accrual that are allowable, since random replacements by transition nucleotides (as opposed to transversions) at the 3^rd^ codon position overwhelmingly (by 94%) favor synonymous substitution. Moreover, a transition at any codon position confers a bias towards both synonymous and conservative amino acid replacement (Tables 4–5, see also [82],[83]). Because the genetic code predated both eukaryotic exons and introns, these findings point to DNA transition bias as a well-organized device that evolved to favor a specific type of DNA variant. The mechanism would act to preserve crucial DNA coding sequences and delimit the types of SNPs and protein polymorphisms most likely to occur.

**Table 4:** Computer Simulation of SNP Accrual in the Setting of a Transition Bias Leads to Enhancement of Synonymous Variants.

Imposed Substitution Bias	Sequence	No. Runs	Muts/Run	Resulting N∶S Ratio	P-Value vs. CFTR unbiased
Unbiased	CFTR	10	50	3.37	----
	CFTR GC-RICH	10	10	4.12	0.018
CFTR Mutation Database Derived Transition Bias (See Table 2)	CFTR	10	50	2.79	7.27 E-81
	CFTR GC-RICH	10	10	3.04	1.29 E-31
Exon Derived Transition Bias (See Table 1)	CFTR	10	50	2.39	1.74 E-222
	CFTR GC-RICH	10	10	2.16	2.74 E-29
Intron Derived Transition Bias (See Table 1)	CFTR	10	50	2.64	1.40 E-121
	CFTR GC-RICH	10	10	2.02	9.50 E-21

SNPs were placed randomly at computer-generated positions in the full-length CFTR sequence, or in a GC-rich region (150 base pair interval (4260–4409) of the human CFTR open reading frame) in an unbiased fashion, or with a transition bias according to the CFTR mutation database (see Table 2), or transition bias observed for either exonic or intronic SNPs from 1000 Genomes (Table 1). GC rich isochores are reported to be more likely sites of natural mutation. The ratio of resulting non-synonymous (N) to synonymous (S) SNPs is shown. The data indicates strong preference for synonymous variants in the setting of transition bias, although magnitude of the effect does not fully account for enhancement of synonymous SNPs shown in Figure 2. Transition bias may therefore represent one (perhaps among several) evolutionary mechanisms serving to augment formation of synonymous DNA polymorphism.

**Table 5:** Computer Simulation of SNP Accrual in the Setting of a Transition Bias Leads to Enhancement of Conservative Mutations.

Imposed Substitution Bias	Sequence	No. Runs	Muts/Run	Resulting Ncon∶Con	P-Value vs. Corresponding unbiased substitution
Unbiased	Artificial Sequence	10	50	2.95	----
	CFTR	10	50	1.93	----
	CFTR GC-RICH	10	10	1.75	----
CFTR Mutation Database Derived Transition Bias (See Table 2)	Artificial Sequence	10	50	2.8	2.46 E-15
	CFTR	10	50	2.04	----
	CFTR GC-RICH	10	10	1.56	2.89 E-19
Exon Derived Transition Bias (See Table 1)	Artificial Sequence	10	50	2.52	5.96 E-58
	CFTR	10	50	1.53	4.31 E-85
	CFTR GC-RICH	10	10	1.57	3.45 E-29
Intron Derived Transition Bias (See Table 1)	Artificial Sequence	10	50	2.25	8.40 E-32
	CFTR	10	50	1.99	----
	CFTR GC-RICH	10	10	1.17	6.49 E-18

SNPs were stochastically placed in 1) an artificial, assembled gene containing 1480 codons arranged randomly (i.e. random codons were used to generate a 4440 bp sequence), 2) the CFTR coding sequence (1480 codons), or 3) a GC-rich region of CFTR. The computer-generated positions to be mutated were selected randomly, and the choice of base replacement (e.g. with or without a particular transition bias) derived as above, according to the CFTR mutation database (Table 2), or rates observed for exonic or intronic SNPs (Table 1). The ratios for non-conservative (Ncon) to conservative (Con) SNPs are shown. Table 5 is the result of 10 simulation runs per sequence, indicating significant differences even after small numbers of SNP incorporation.

In unpublished studies, we recently compiled a genomic analysis of sixteen distinct strains of *Mus musculus* (http://www.sanger.ac.uk/cgi-bin/modelorgs/mousegenomes/snps.pl) [74]. We found that high prevalence DNA motifs surrounding single nucleotide polymorphisms were markedly underutilized by mammalian anticodons (Plyler, *et al.*, manuscript submitted). Because the same SNP promoting elements were otherwise conserved across murine exons, introns, and intergenic regions, this result could not manageably be attributed to ongoing purifying selection at the level of protein function. Instead, the data suggested an exon-sparing **mechanism** that regulates SNPs at the time of their formation and serves to minimize mutations within exonic DNA. The pathway was best interpreted as another device that co-evolved with both the genetic code and codon usage to help preserve the exome of higher organisms.

### Intronic DNA as an exon-sparing mechanism

In contrast to exonic DNA, significant numbers of intronic and intergenic SNPs are already known to be generated in a constrained and arguably predictable manner based on transition bias, proximity to DNA recombination sites, nucleosome structure, GC rich isochores, or specific sequence contexts [10], [76]–[82]. Non-coding SNPs provide genomic and phenotypic variation (through modification of crucial regulatory elements, microRNAs, expressed noncoding sequences, etc.) without the need for substantial transmutation of the open reading frames. Based on the analysis presented here and the vital imperative to avert “meltdown” of protein coding DNA, we speculate that the introme, itself, might not only serve as an evolutionary strategy to support the generation of diversity (through alternative splice variants, microRNAs, gene network regulation, etc.), but as a specific alternative to meddling with the exons. Non-coding DNA in this model would appear quite expendable for a given species over a few generations (as true for CF mice, innumerable other transgenic animals in which non-coding DNA has been disrupted to delete, insert, repair, or select for gene modifications, or animals in which large expanses of the non-coding genome have been intentionally omitted without phenotypic effect [83]), but would be essential for selfish genomes attempting to cope with environmental challenge over an evolutionary timeframe. If the noncoding compartment originated in part as a strategy that helps DNA more safely ‘experiment’ with its own diversity (without running the risk of “meltdown”), the metabolic expense of intronic elements might be partly justified on that basis alone. In principle, by setting conservative limits regarding: 1) the range of single nucleotide mutation rate, 2) extent to which random coding mutations are expected to disrupt protein function, and 3) likelihood that SNPs in non-coding DNA would alter gene expression, simulations to evaluate this hypothesis might be more formally undertaken in the future.

### Relevance to population-type analysis

The results presented in this report should be viewed in light of earlier, population studies regarding natural selection and the consequent distribution of SNP fitness effects. For example, our finding of synonymous SNP enrichment agrees with previous data in *Drosophila*, human, and other species, including seminal observations from Kreitman and Colleagues [35]–[37], [61], [84]–[85]. On the other hand, earlier work applying data of this sort to a Poisson-type random field model in human populations has led to conclusions different from those described here [36], [84], [86]. For example, Boyko and colleagues [84] investigated genomic SNP ratios and reported a synonymous∶non-synonymous frequency of 1.38 among Caucasians (very similar to the value of 1.3–1.6 shown in Figure 2). When a mathematical treatment was conducted (based in part on earlier quantitative work in *Drosophila* [87] and human [88]), the data fit best to a distribution in which approximately 30% of random, non-synonymous single base replacements were suggested to be highly deleterious ([84]; a finding quite different from our analysis). A study by the same group used similar quantitative methods to test natural selection among conserved non-coding elements, and concluded that synonymous SNPs in human genes are under strong (recent) positive selective pressure [86]. We note that either of these interpretations might be significantly influenced by a preference towards synonymous SNP formation. Although Poisson field experiments have utilized the best available demographic parameters, such studies require a neutral set of variants (typically synonymous SNPs) against which evolution of specific genes, genetic elements, or proteins at selected sites can be ratioed or otherwise compared [89]. Our findings suggest a strong non-neutral component during the production of new synonymous or other SNPs. We therefore believe future analyses might benefit from more prominently considering the contribution of synonymous SNP formation bias as described by the present report.

In this study, we also considered more classical, population-based analysis of natural selection utilizing McDonald-Kreitman methodology to test human CFTR variants from over 1,000 individuals and numerous ethnicities (1000 Genomes), and compared this data to an outgroup representing the most recent chimpanzee sequence (CHIMP 2.1.4) The proportion of base substitutions fixed in human versus differences between human and chimp indicated α = (−) 2.72, with neutrality index of 3.72 (uncorrected p value = 0.07). Based on classical interpretation, our findings could be taken to suggest a trend towards positive CFTR selection with non-neutral divergence for the human CF gene. On the other hand, evidence presented here indicates synonymous SNP formation is often non-neutral due to features such as transition bias. This aspect complicates a conventional assessment of selection intensity, which (as above) typically requires a well-defined cohort of random and fitness neutral (e.g. synonymous) SNPs for comparison to non-synonymous variants. For example, we show that CFTR (like many other genes) exhibits discrete exonic regions with high CpG content (Tables 4–5). Such coding intervals represent sites for augmented DNA methylation, increased transition bias and enhanced synonymous SNP formation. Awareness of such domains and their quantitative significance may influence the interpretation of selection intensity, including gene segments believed to undergo rapid evolution, which are otherwise predicated on largely random (and neutral) formation of non-coding or synonymous SNPs.

### Concluding remarks: an adaptation to enhance adaptability

In this report, we interpret proteins and their constituent exons as ‘lessons’ of inestimable value picked up from iterative attempts at long term DNA survival. We maintain that despite the need for variation, lessons such as these should be viewed as far too valuable to expend—particularly when alternatives such as non-coding DNA might be utilized instead. We suggest that SNP formation in CFTR and other human genes appears configured to help preserve exons (e.g. with the majority of SNPs adhering to a set of rules that strongly bias their formation as intronic, synonymous, contextual, transitional etc.), constrained by specific molecular mechanisms such as those involving transition bias and codon usage, and should therefore be viewed as meaningful and adaptive. A precedent for the sort of adaptation proposed here has already been described for a different evolutionary mechanism, the combinatorial immune system, where antediluvian trial and error turned up a highly organized means of generating extensive diversity that is capable of responding to infectious agents not yet encountered by a species. We note that the adaptive immune response provides remarkable phenotypic variation that is regulated, has worked well through the ages, is readily explained without evoking directional evolution [24], [72] or intelligent design [73], and is undoubtedly based on multiple earlier prototypes that failed or were much less effective. In the same fashion, we suggest that adaptive pathways have evolved to help regulate DNA diversification. Such mechanisms could have appeared, for example, after countless failed attempts at long term DNA survival that ended in unchecked SNP accumulation and genomic meltdown.

In summary, more attention should to be paid to ways in which production of human DNA polymorphism (i.e. at the time of SNP formation) is organized. A review of quotidian sequence data provided throughout this report indicates that genome-wide SNP patterns should be evaluated in light of long-term DNA survival and modern knowledge regarding protein plasticity. If HapMap, 1000 Genomes, and related projects in other species increasingly reveal predictable or mechanistically relevant patterns of SNP distribution as contemplated by the present study, evolution might be viewed as more regulated than has been classically interpreted. This report therefore suggests the existence of mechanisms by which DNA may regulate its own diversity, safeguard the hard-earned lessons encoded by genes, and help guide its own evolutionary path.

## Materials and Methods

### Exonic and synonymous SNP frequencies identified by 1000 Genomes

SNPs in 133 human genes known to be lethal or severely debilitating when deleted [90] or 4857 human genes for which intron/exon boundaries are readily definable in the Exon-Intron Database (http://www.utoledo.edu/med/depts/bioinfo/database.html), and a 1000 Genomes release (http://pilotbrowser.1000genomes.org/index.html; 6 individuals of European or African descent) were evaluated. The length of each coding sequence and the combined lengths of the intronic sequences (Exon-Intron Database), together with SNP information (1000 Genomes) were obtained. SNP totals were normalized to numbers of exonic or intronic nucleotides (exonic and intronic SNP percentages, respectively).

For evaluation of synonymous versus non-synonymous mutations, two datasets were used: SNPs in 98 of the 133 genes described above with at least one exonic variant, and 13,820 genes for which data was accessible (from http://www.utoledo.edu/med/depts/bioinfo/database.html and 1000 Genomes). Synonymous and non-synonymous SNPs were normalized to total exonic nucleotide content as above.

As a test of SNP authenticity, we manually inspected a random population of 200 coding and non-coding variants selected from 1000 Genomes using Interactive Genome Viewer (IGV) software. We tested these for features shown previously to indicate sequencing error, and found less than 2–3% of SNPs exhibited low quality score, inconsistent consensus, misalignment, artifactually high “coverage” (“pile-up”) due to homologous sequences elsewhere in the genome, duplicated reads, indels, short local repeats, etc. This result provided independent confirmation for robustness of SNP data available from the 1000 Genomes resource.

### HapMap based identification of minor allelic frequencies for CFTR and NF1 across four major ethnicities

Frequency data for SNPs in CFTR or NF1 were collated for each of four ethnicities: JPT (Japanese in Tokyo, 45 individuals); CHB (Han Chinese in Beijing, 45 individuals); CEU (or CEPH, Utah residents with ancestry from northern and eastern Europe, 90 individuals); and YRI (Yoruba in Ibidan, Nigeria, 90 individuals) using HapMap (http://hapmap.ncbi.nlm.nih.gov/). Minor allelic frequency (MAF) refers to the relative frequency (1000 = 100% incidence) of the minor allele at each SNP position. We focused our attention on SNPs verified by HapMap and with minor allelic frequency greater than zero in at least one population (i.e. at least one individual among 270 in the database exhibited the minor SNP). This convention allowed us to use HapMap as a less biased tool for cataloging genomic variation among very diverse individuals. In order to generate a MAF block diagram, allele frequency data was downloaded from the UCSC genome table browser (http://genome.ucsc.edu/cgi-bin/hg:tables). After downloading, SNPs with MAF equal to zero among all four ethnicities were omitted to enhance stringency. The remaining SNPs were then utilized to generate scatter plots.

### Analysis of transition SNPs and Y chromosomal SNPs in human genes

SNP configurations (transition and transversion) for intronic and exonic regions using the set of 98 human genes described above with at least one exonic SNP were collated. Frequencies of CFTR SNP subtypes (transition vs. transversion) among>1000 SNPs, many of which have been implicated in clinical CF, were also tallied (http://www.genet.sickkids.on.ca/cftr/app). SNP totals for the Y and other chromosomes were obtained directly from dbSNP, HapMap, and 1000 Genomes, and presented in tabular form.

### Simulation of mutation accrual in the setting of a transition bias

SNPs were placed at computer-generated positions in 1) the full-length CFTR sequence, 2) a GC-rich region of CFTR (150 base pair interval (4260–4409) of the open reading frame), or 3) an artificial, assembled gene containing 1480 codons arranged randomly (i.e. random codons to generate a 4440 bp sequence).

The site for each base substitution was determined by a random number function that served as an index into an array representing the desired base substitution ratio. For example, an unbiased base substitution array for the replacement of adenine contained cytosine, guanine and thymine in equal proportions, so that these were chosen with equal probability to replace adenine. The base substitution ratios were:

1. Unbiased – each of the four bases was replaced with equal probability by any of the other three.
2. CFTR Mutation Database – All entries in this database (http://www.genet.sickkids.on.ca/cftr/app) describe directional substitution, such as A to C, and were used to calculate base substitution frequencies.
3. Exon or Intron Base Substitution Ratios – were obtained as above from exons of 98 human genes or CFTR introns. Because the directionality of mutation is often not known (i.e. for a SNP encoding A or T, it is unknown whether A→T or T→A), and since 1) the directionality is not relevant to this particular test of transition bias, and 2) there is clearly a transition bias regardless of directionality judged by studies of more recent mutations in CFTR, the incidences of both directions were analyzed together.

Each simulation run imposed 10–50 base replacements on the human CFTR coding sequence or the artificial sequence, and 10 runs were conducted for each condition. In all instances, a site (base) was allowed to mutate more than one time, although in practice this seldom occurred. Due to the comparatively short length of the GC-rich region, 10 mutated sites were tested per simulation. Results from simulations were determined following translation and alignment with the authentic sequence.

A modified version of the above algorithm was established to model the long term consequence of mutation accrual in CFTR. Similarity to an original CFTR sequence was evaluated by translation of the resulting cDNA after several thousand generations in order to evaluate protein integrity.

Code for the above simulations was designed as follows:

1. A sequence related to CFTR as described above was represented as a character array (DNA bases: A, C, G,T).
2. Base replacement arrays were populated to reflect the particular substitution bias being simulated. For example, Table 2 lists 1036 CFTR SNPs found to be associated with human disease. From these, replacement frequency for each of the possible substitutions was calculated, and used to model SNP accrual as described below.
3. The particular position to be mutated was selected by a random number within the range of the length of the sequence to be mutated. This random number was used as an index into the array from Step 1.
4. The sequence to be mutated was read as an array using the random number from Step 3. The base at that position was determined.
5. For example, if position 5 of the sequence to be mutated contained an A, the A-substitution array (Step 2) would be consulted to determine the replacement base. A random number determined which of the three bases would replace the A. The random number was used to index the A-substitution array created and populated in Step 2.
6. The position in the sequence to be mutated randomly selected in Step 3 was overwritten with the replacement base from Step 5.
7. When the desired number of simulated mutations had been completed, the mutated sequence codons were compared with corresponding original sequence to determine the numbers of synonymous or non-synonymous mutations and (for the non-synonymous mutations) the numbers of conservative versus non-conservative amino acid replacements.

### SNP concordance among evolutionarily distant species

CFTR and 21 other genes with ≥50% concordance across six non-human species (rat, mouse, dog, opossum, chicken, and frog) were obtained from the UCSC genome browser. Sequences of the non-human species were aligned using the ClustalW2 tool (http://www.ebi.ac.uk/Tools/clustalw2/index.html). The number of bases identical among the non-human species was divided by the largest total number of bases in any species to obtain the most conservative measure of percent concordance. Locations of exonic human SNPs in each gene were obtained from 1000 Genomes. Following alignment of all six non-human species, the variance among non-human genes was examined using human SNPs as a marker. For example, after observing a SNP in human CFTR at nucleotide position 593 and the corresponding position in six other species, a determination was made as to whether or not the non-human species also exhibited polymorphism at that site.

In addition, CFTR homologs in four evolutionarily distant species (horse, frog, zebrafish, and shark) were aligned with the human coding strand, both independently and collectively. In the collective alignment, ∼43% of the coding sequence was invariant. A computer simulation was conducted and the total number of differences from human placed randomly within the human CFTR reading frame of 4443 bp. This simulation was performed 120,000 times, and the total number of differences from human (distributed as random point mutations) was tabulated. The goal was to determine whether concordance observed in a multiple species alignment could be accounted for by chance.

### Statistical analysis

Comparison for SNP frequencies (exonic versus intronic, synonymous versus non-synonymous, transition versus transversion, observed versus predicted computer simulation frequencies, etc.) were conducted by χ^2^ and contingency table analysis (2×2 tables employing Yates' correction for continuity).

## Supporting Information

Figure S1 **Minor allelic frequencies of prominent CFTR SNPs.** The heat map emphasizes findings shown in Figure 3A; i.e. much higher minor allele frequencies (MAFs) among SNPs in CFTR for CEU, CHB, and JPT, compared with YRI. When MAFs were plotted against physical location, haplotype blocks were evident in three of four ethnicities, and underrepresented in the ‘back’ half of CFTR. The observation is attributable to a greater number of YRI ancestral haplotypes, additional cross-over events among YRI, higher numbers of SNPs formed since founding of YRI, or some combination of these factors. The YRI ethnicity exhibited no MAF block structure or positional enhancement of variation, in contrast to other ethnic groups.
(TIF)

Table S1 **SNP incidence in human intronic and exonic DNA.** SNPs in 133 human genes known to be lethal or severely debilitating if deleted [90].
(TIF)

Table S2 **Synonymous and non-synonymous SNP incidence.** Exonic SNPs in 98 of the 133 genes shown in Figure 1A, specifically those containing at least one exonic SNP.
(TIF)

Table S3 **Relationship between human SNPs and variable nucleotide positions of non-human species.** From among 98 genes described in Figure 2A and Table S2, those with at least 50% concordance among six nonhuman species (rat, mouse, dog, opossum, chicken, frog) were selected for further analysis (22 genes total). The number and type of exonic SNPs in human (from 1000 Genomes) and correspondence with known polymorphic regions in other vertebrate species is shown.
(TIF)
